# A Water Cluster Conduit in Crystal

**DOI:** 10.3390/molecules22122278

**Published:** 2017-12-20

**Authors:** Fangfang Jian, E Liu, Jiao Xu

**Affiliations:** School of Chemical Engineering and Pharmaceutics, Henan University of Science and Technology, Luoyang 471023, China; 798233143@haust.edu.cn (E.L.); 18437960281@163.com (J.X.)

**Keywords:** glycine, water cluster, nanometer sized, crystal, hydrogen-bond

## Abstract

The crystal structure of compound (**1**), [CuCl(phen)(H_2_NCH_2_COO)]∙4H_2_O, reveals an unusual hydrogen-bond water cluster aggregate T6(2)6(2). Four water molecules in (**1**) form an isolated water cluster, [(H_2_O)_14_]_n_, resembling a “phenanthro-[1,2]phenanthrene polymer structure shape” along the *ac* plane. The two face-face parallel [(H_2_O)_14_]_n_ planes are bridged by Cl atoms in [CuCl(phen) (H_2_NCH_2_COO)] with a strong O-H∙∙∙Cl hydrogen bond to form a water cluster conduit.

## 1. Introduction

Water is the most abundant compound on Earth, and is very difficult to eliminate from other solvents (even when there is no water in the synthesis steps). Furthermore, it is not present in the solvents used for crystallization where hydrates can be obtained. The presence of water molecules in any crystal structure can play an important role in stabilizing some supramolecular species, as the number of hydrogen bond acceptors and donors can differ from those of anhydrous compounds [1,2]. Structural studies on discrete water clusters within the lattice of a crystal host have significantly advanced our understanding of the behavior of bulk water [3,4,5,6]. Elucidating the assembly of discrete water aggregates, and understanding how water aggregates interact with a crystal host, are still challenging scientific endeavors [7,8,9,10,11,12,13]. A number of water oligomers have been extensively studied using X-ray diffraction methods [14,15,16,17,18,19,20,21,22,23,24]. The aggregation of lattice water molecules in crystals, into hydrogen-bonded clusters as well as infinite networks, has generated considerable interest [25,26,27,28,29,30,31,32,33,34]. Infantes et al. [35,36] examined about 1400 hydrated structures, retrieved from the Cambridge Structural Database (CSD), and performed classifications of water/water motifs, using statistics regarding their relative frequencies. The established classification system is as follows: D (discrete chain), R (discrete rings), C (infinite chains), T (infinite tapes), and L (infinite layers). In Reference [37], a proper analysis was lacking, which should be done when reporting any new water morphologies in a crystal lattice. With this in mind, and with the help of the Cambridge Crystallographic Data Centre (CCDC), we performed a Cambridge Structural Database (CSD) search for the phenanthrene structure-shaped motif with 14 water molecules, of which the aggregate is T6(2)6(2). We did not find any phenanthrene structure-shaped patterns in CCDC. Therefore, there is presently no structural information available for a discrete, phenanthrene structure-shaped (H_2_O)_14_ cluster, nor for its participation in the stabilization of a suramolecular assembly. In this paper, we report on an unusual hydrogen-bond water cluster, [(H_2_O)_14_]_n_, which can form nanometer-sized water cluster conduits (with a diameter of about 12.034 Å) using a strong O-H∙∙∙Cl hydrogen bond.

## 2. Results

CuCl_2_·2H_2_O (1.70 g, 0.01 mol) was added to a heat solution (80~90 °C) of glycine (H_2_NCH_2_COOH) (0.75 g, 0.01 mol) and NaOH (0.40 g, 0.01 mol) in 40 mL of water. The reaction mixture was stirred at about 90 °C for 10 min. Then phenanthroline (3.55 g, 0.02 mol) was added, and the solution was stirred for another 4~5 h at about 90 °C. After filtering out the precipitate, the resulting solution was left to stand undisturbed. Upon slow evaporation at room temperature, blue crystals were obtained from the mother liquor. They were all collected, dried, and submitted for elemental analysis. The compound (**1**) was achieved with a yield of 75%. The single crystals X-ray confirmed that the blue crystal was [CuCl(phen)(H_2_NCH_2_COO)]∙4H_2_O (**1**). The elemental analysis supports this formulation.

**Figure 1:**
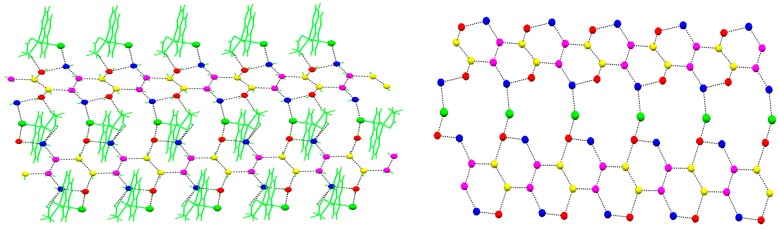
(**Left**) Depicting the hydrogen bond interaction of [CuCl(phen) (H_2_NCH_2_COO)] with the water cluster; (**Right**) View of the water–chloride T12(3) aggregate. The water clusters and Cl atoms are depicted as a ball and stick model for clarity. Colors are as follows: blue, O1w; red, O2w; pink, O3w; yellow, O4w; black dashed, hydrogen bond; green and capped stick, [CuCl(phen)(H_2_NCH_2_COO)] group and hydrogen atom.

**Figure 2:**
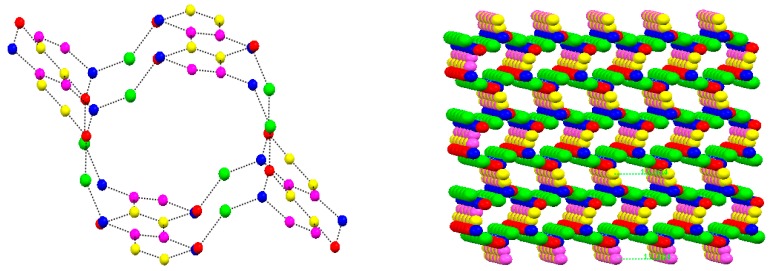
(**Left**) The formation of the water cluster cavity; (**Right**) View of the nanometer-sized water cluster conduit in the *a* axis. The Cl atoms are detained and [CuCl(phen) (H_2_NCH_2_COO)] is omitted for clarity. Colors are as follows: blue, O1w; red, O2w; pink, O3w; yellow, O4w; Green, Cl atom.

Figure 1 shows the hydrogen bond interaction between [CuCl(phen) (H_2_NCH_2_COO)] and the water cluster [(H_2_O)_14_]_n_, and view of the water-chloride T12(3) aggregate. Figure 2 shows the nanometer-sized water cluster conduit. Table 1 and Table 2 list the hydrogen bonds and π-π interactions, respectively.

## 3. Discussion

The compound (**1**) contains one [CuCl(phen) (H_2_NCH_2_COO)] and four water molecules. There is no obvious bonding interaction between the [CuCl(phen)(H_2_NCH_2_COO)] groups with the shortest distance of Cu∙∙∙Cu 7.010(1) Å (Figure 1, left). The coordination environments of each Cu(II) atom is pentacoordinated with three N atoms from phenanthroline ligands and NH_2_ of glycine, one O atom from carboxy of glycine, and one Cl atom in a distorted square pyramidal geometry. Three nitrogen atoms and one oxygen atom occupy the basal sites with Cu-N distances of 2.000(5)~2.028(5) Å and Cu-O distances of 1.950(4) Å. The coordinated chloride occupies the apical position with Cu(1)-Cl(1) distances of 2.577(2) Å. The bite angle of chelating glycine and phenanthroline ligands O(1)-Cu(1)-N(3) and N(1)-Cu(1)-N(2) is 83.8(2)° and 81.9(2)°, respectively. The Cu atom with the phenanthroline ligand are quite planar, and the maximum atom deviation from the least squares plane is 0.039(3) Å. The glycine ligand with the Cu atom is nearly planar, with the largest deviation of 0.203(1) Å for O(2). The dihedral angle between the glycine moiety and phenanthroline group is 11.58°. In the crystal building, the [CuCl(phen)(H_2_NCH_2_COO)] is held together by means of face-to-face *π-π* interactions among the aromatic phenanthroline ligands and a glycine Cu five-membered chelating ring to form layers parallel to the *ac* plane.

It is especially interesting to note that four water molecules in compound (**1**) form a water cluster, [(H_2_O)_14_]_n_. To best our knowledge, this T6(2)6(2) aggregate represents a new water cluster structure, which has not been reported before [35,36,37]. The water cluster [(H_2_O)_14_]_n_ units are strongly held together by O-H∙∙∙O interactions with the O∙∙∙O distance ranging from 2.408 to 2.755 Å (average: 2.612 Å) and the O∙∙∙O∙∙∙O angle ranging from 98.80° to 133.00° (average: 117.58°). These values are comparable to the values in ice *I_h_* [38]. The water molecules O3w and O4w on the C2 symmetry create C4 infinite chains, and the O1w and O2w are linked to O3w and O4w molecules, respectively, to form a “phenanthrene structure shape”. There is a pair of independent water cluster polymers in compound **1**. Each water layer is almost planar, with the largest atom deviation from the least squares plane of water cluster being 0.736 Å for one and 0.747 Å for the other, respectively (Supplementary Materials Figure S5). The dihedral angle between the two adjacent water cluster planes is 30.50°. The distances of the parallel water cluster plane are about 12.0 Å (Supplementary Materials Figure S6). It should be noted that the isolated water cluster [(H_2_O)_14_]_n_ forms a similar geometry configuration to that of “phenanthro-[1,2]phenanthrene polymer”. Also, there are three longer bonds (2.755, 2.753, and 2.742 Å) and three shorter bonds (2.408, 2.410, and 2.607 Å) in the hexamer unit.

The two adjacent infinite water cluster polymers, [(H_2_O)_14_]_n_, are linked by a strong O-H∙∙∙Cl hydrogen bond [39]. The Cl atom in the [CuCl(phen) (H_2_NCH_2_COO)] group joins the two adjacent water clusters with the distance of Cl∙∙∙O1w 3.080 Å and Cl∙∙∙O2w 3.223 Å, and the angle of O1w∙∙∙Cl∙∙∙O2w 48.85°, respectively (Figure 1, right). If we consider an extended motif, the four water molecules (2O1w, O2w, O3w, and O4w) in the T6(2)6(2) water aggregate and chloride generates a cyclic motif T12(3). These water-chloride aggregate to join the two face-face parallel infinite water cluster polymers, forming a nanometer-sized water cluster conduit (Figure 2). The diameter of the water cluster conduit is about 12.0 Å. The strong hydrogen-bonding ability of the Ow∙∙∙Cl linker employed here plays a crucial role in promoting and stabilizing the aggregation of the water channel, thereby leading to the infinite nanometer-sized water cluster conduit in the crystal structure. 

There are some intermolecular hydrogen bonds between the amino-group and carboxy group in [CuCl(phen)(H_2_NCH_2_COO)] and water clusters [(H_2_O)_14_]_n_. The donor and acceptor distances are O1w∙∙∙O1 2.921 Å, O1w∙∙∙O2 3.005 Å, and O2w∙∙∙N3 3.043 Å, respectively. Compared with the interior hydrogen bond in water cluster polymers, the exterior ones are easily separated, which reflects the relatively weak interactions. The hydrogen-bonding parameters give more evidence about the stability of water cluster polymers. The average Ow∙∙∙Ow separation of 2.612 Å in a water cluster polymer is significantly shorter than the average O∙∙∙Ow and N∙∙∙Ow separation of 2.990 Å.

**Figure 3:**
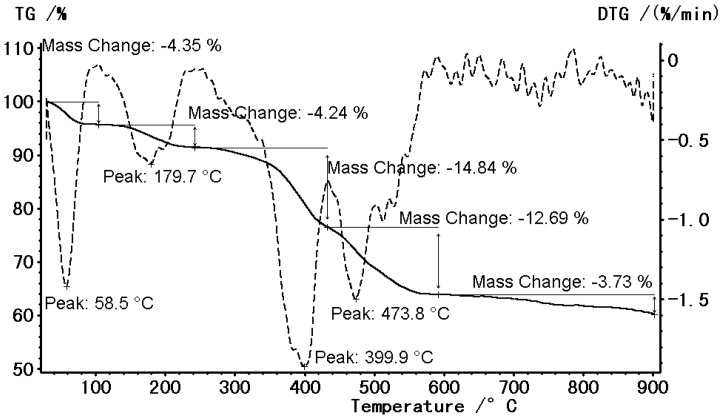
TG and DTG analysis for compound (**1**).

To gain more insight into the properties relative to water clusters, the dehydration behavior of compound (**1**) was investigated using thermogravimetric analysis. The TG and DTG curves of compound (**1**) are shown in Figure 3. The first step (58 °C–80 °C) corresponds to the loss of one water molecule with the heat-absorption peaks (found 4.35% calc. 4.23%). The second step, occurring between 160 °C and 260 °C, corresponds to the loss of another water molecule (found 4.24% calc. 4.23%). The third step, between 300 °C and 400 °C, corresponds to the decomposition of the other two water molecules and the anion H_2_NCH_2_COO^−^. The residual compound is perhaps CuCl(phen)(HCOO) (found 14.84% calc. 14.34%). Above 450 °C, there are larger errors between the calculated values and the tested values, which, on the one hand, may be due to experimental error, and on the other hand, may be due to the decomposing debris of molecules. 

## 4. Materials and Methods 

The C, H, and N elemental analyses were performed on a Perkin-Elmer elemental analyzer. Crystals data were collected on an Enraf-Nonius CAD-4 diffractometer with graphite monochromated Mo *K_α_* radiation (λ = 0.71073 Å). Intensities were corrected for Lorentz and polarization effects and empirical absorption, and data reduction was carried out. The structure was analyzed by the direct method. These data can be observed at the Cambridge Crystallographic Data Center via www.ccdc.cam.ac.uk/data-request/cif. The CCDC number is 644280.

A typical experimental procedure for compound (**1**) is below: Cupric chloride, sodium hydroxide, H_2_NCH_2_COOH, phenanthroline, and other chemical reagents were obtained from commercial sources and used without further purification. To a 100-mL flask, 0.01 mol of CuCl_2_∙2H_2_O (1.70 g), 0.01 mol of NaOH (0.40 g), and 0.01 mol H_2_NCH_2_COOH (0.75 g) in 40 mL of deionized water, as well as 0.20 mol (3.55 g) of phenanthroline in 20 mL of ethanol was added while stirring at a temperature 80~90 °C. The reaction was maintained for 4~5 h until the solution to clarification, following which the solution was filtered. After filtering out the precipitate, the resulting solution was allowed to stand at room temperature. Deep blue single crystals suitable for X-ray measurements were obtained over one week. From the element analysis below and the single crystal X-ray, we concluded that the compound (**1**) is [CuCl(phen)(H_2_NCH_2_COO)]∙4H_2_O. Anal. Calc. for C_40_H_56_Cl_2_Cu_2_N_8_O_13_: C, 39.50%; H, 4.70%; N, 9.87%, Cu; 14.93%, Cl, 8.35%; O, 22.57%. Found: C, 39.32%; H, 4.80%; N, 9.68%.

## 5. Conclusions

In summary, a water cluster conduit in [CuCl(phen)(H_2_NCH_2_COO)]∙4H_2_O crystal was assembled. The water cluster aggregate mode, T6(2)6(2), and water-chloride extended aggregate mode, T12(3), have not been predicted theoretically nor previously reported experimentally. We also found that this structure of water cluster polymer was much like that of the phenanthro-[1,2]phenanthrene polymer in configuration. Water molecules, which have two hydrogen atoms and two lone pairs enabling them to participate in four hydrogen bonds in a tetrahedral arrangement, may be comparable with carbon *sp*^3^ covalent chemistry, and form various geometry structures, similar to the carbon atom. This new structural data definitely enhances the understanding of the one-dimensional and two-dimensional structural aspects of water.

## Figures and Tables

**Table 1:** Geometrical parameters of hydrogen bonds (Å, deg) for the water cluster.

Length		Angle	
O1w-O1	2.920(1)	O2w ^F^…O1w…O3w ^C^	114.39
O1w-O2	3.005(1)	O2w ^F^…O1w…O1	84.88
O1w-Cl(1) ^A^	3.080(2)	O2w ^F^…O1w…O2	109.56
O1w-O3w ^C^	2.757(1)	O2w ^F^…O1w…Cl(1) ^A^	99.34
O2w-O1w ^B^	2.743(1)	O3w ^C^…O1w…O1	96.28
O2w-O4w ^C^	2.754(1)	O3w ^C^…O1w…O2	115.53
N3-O2w ^C^	3.043	O3w ^C^…O1w…Cl(1) ^A^	126.05
O2w ^B^-Cl(1)	3.223(1)	O1w ^B^…O2w…O4w ^C^	98.83
O3w-O4w	2.403(1)	O1w ^B^…O2w…N3 ^C^	94.22
O3w-O3w ^D^	2.412(1)	O1w ^B^…O2w…Cl(1) ^B^	103.79
O4w-O4w ^E^	2.608(1)	O4w ^C^…O2w…N3 ^C^	91.39

Symmetry code: ^A^, −*x*, −1/2 + *y*, 1/2 − *z*; ^B^, 1 + *x*, *y*, *z*; ^C^, 1 − *x*, −1/2 + *y*, 1/2 − *z*; ^D^, 1 − *x*, 1 − *y*, −*z*; ^E^, −*x*, 1 − *y*, *z*; ^F^, −1 + *x*, *y*, *z*.

**Table 2:** π-π interactions (face-to-face) in complex (**1**) ^a^.

Ring(*i*)→Ring(*j*)/C	Distance between the (*i,j*) Ring Centroids (Å)	Dihedral Angle (*i,j*) (Deg)	Distance of Centroid (*i*) from Ring (*j*) (Å)
R1**→**R4 ^i^	3.528	0.67	3.354
R2**→**R3	3.618	2.10	3.379
R2**→**R4 ^ii^	3.844	0.92	3.372
R2**→**R4 ^i^	3.588	0.92	3.379
R4**→**R4 ^ii^	3.528	0.67	3.389
R4**→**R4 ^i^	3.865	0.00	3.389

^a^ Symmetry code: (i) = −*x*, 1 − *y*, 1 − *z*; (ii) = 1 − *x*, 1 − *y*, 1 − *z*. R(*i*)/R(*j*) denotes the *i*th/*j*th rings of phen: R(1) = Cu(1)/N(1)/C(12)/C(11)/N(2); R(2) = N(1)/C(1)/C(2)/C(3)/C(4)/C(12); R(3) = N(2)/C(10)/C(9)/C(8)/C(7)/C(11); R(4) = C(4)/C(5)/C(6)/C(7)/C(11)/C(12).

## Supplementary Materials

The supplementary materials are available online.
