# Supplementary material for: A Water Cluster Conduit in Crystal
Source: Molecules. 2017 Dec 20;22(12):2278. doi: 10.3390/molecules22122278 (PMC6150000; doi:10.3390/molecules22122278)
Supplement: Supplementary file 1 [file molecules-22-02278-s001.pdf]

Supplementary Materials

A Water Cluster Conduit in Crystal

Fang Fang Jian, E Liu, Jiao Xu

School of Chemical Engineering and Pharmaceutics, Henan University of Science and Technology, Luoyang, Henan, P. R. China. E-mail: ffj2013@163.com

Table of contents **Page**

1. General **S-1**

2. Perspective View **S-2 ~ S-4**

3. Crystallographic data for the compound **S-5**

4. Table of bond lengths and angles **S-6**

5. Table of Geometrical Parameters of Hydrogen Bonds for the Water Cluster **S-7**

6. Table of π-π Interactions **S-8**

1. General

The C, Hand N elemental analyses were performed on a Perkin-Elmer elemental analyzer. Crystals data were collected on an Enraf-Nonius CAD-4 diffractometer with graphite monochromated Mo K_{α} radiation (λ = 0.71073Å). Intensities were corrected for Lorentz and polarization effects and empirical absorption, and the data reduction was carried out using SADABS program. The structure was solved by direct methods using SHELXS-97. All the non-hydrogen atoms were refined on F^2 anisotropically by full-matrix least squares method. The hydrogen atom positions were fixed geometrically at calculated distances and allowed to ride on the parent carbon atoms. Atomic scattering factors and anomalous dispersion corrections were taken from International Table for X-Ray Crystallography. Summaries of crystal and intensity collection, and bond distances and angles of the compounds are given.

A typical experimental procedure for compounds **1** are below: Cupric chloride, sodium hydroxide, glycine, phenanthroline and other chemical reagents were obtained from commercial sources and used without further purification. To a 100 mL flask 0.01 mol of $CuCl_2{\cdot}2H_2O$ (0.17 g), 0.01 mol of NaOH (0.04 g) and 0.01 mol glycine (0.08 g) in 40 mL of deionized water, 0.25 mol (0.50 g) of phenanthroline in 20 mL of ethanol was added with stirring at temperature 80 ~ 90°C. The reaction was maintained 4 ~ 5 hours until the solvent was turned to clarify, and then was filtered. After filtering out the precipitate, the resulting solution was allowed to stand at room temperature. The deep blue single crystals suitable for X-ray measurements were obtained for a week. Yield: 70% (bases on cupric chloride, $CuCl_2{\cdot}2H_2O$). From the element analysis below and the single crystal X-ray, we conclude the compound **1** is $[CuCl(phen)(H_2NCH_2COO)]{\cdot}4H_2O$. Anal. Calc. for $C_{40}H_{56}Cl_2Cu_2N_8O_{13}$: C, 39.50%; H, 4.70%; N, 9.87%, Cu; 14.93%, Cl, 8.35%; O, 22.57%. Found: C, 39.32%; H, 4.80%; N, 9.68%.

2. Perspective View

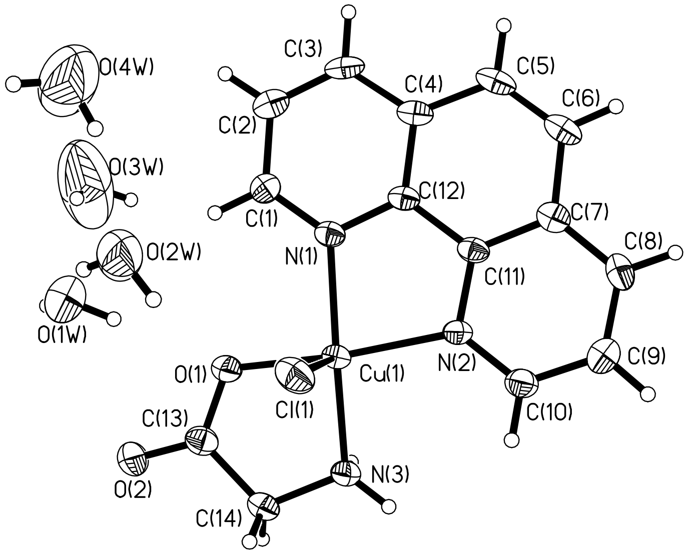

Fig. S1. ORTEP drawing of structural unit

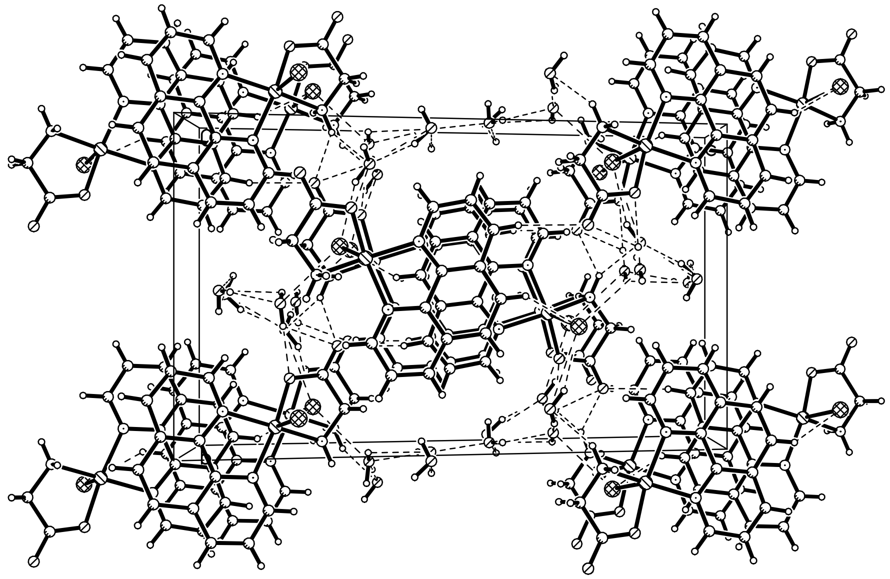

Fig. S2. The packing diagram of the complex viewed along *a* axis

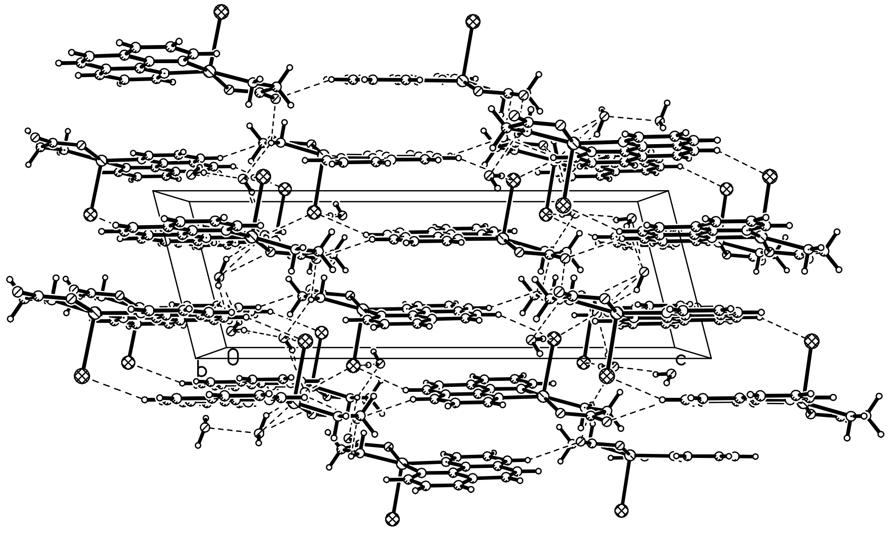

Fig. S3. The packing diagram of the complex viewed along *b* axis

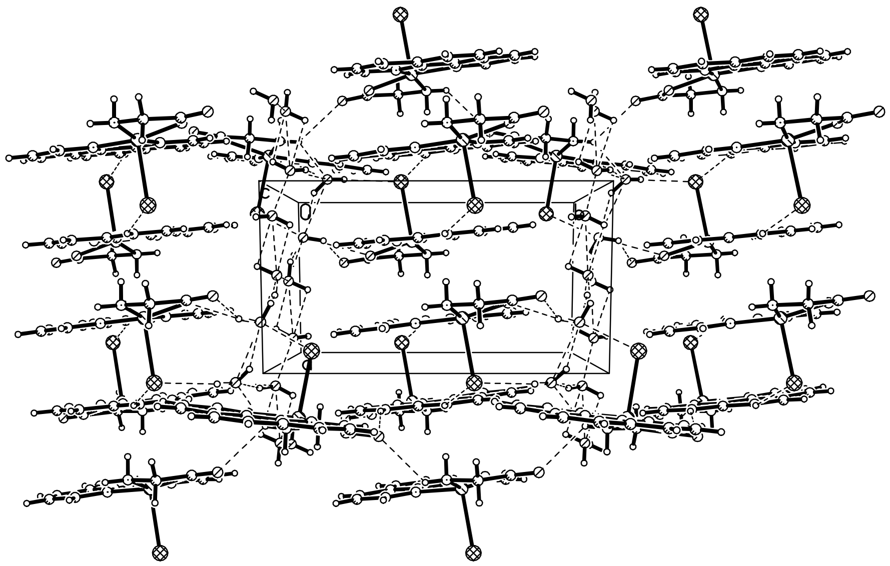

Fig. S4. The packing diagram of the complex viewed along *c* axis

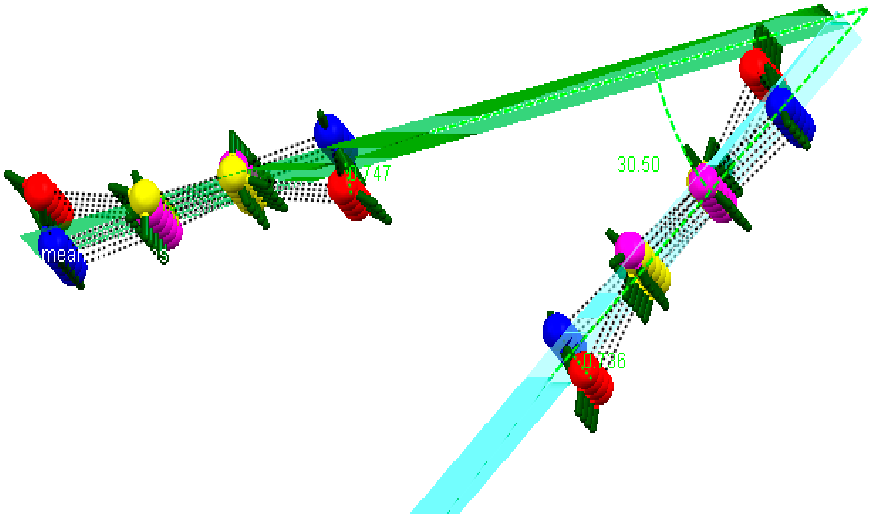

Fig. S5. The dihedral angle between the two adjacent water cluster planes and the largest atom deviation from the mean water cluster plane.

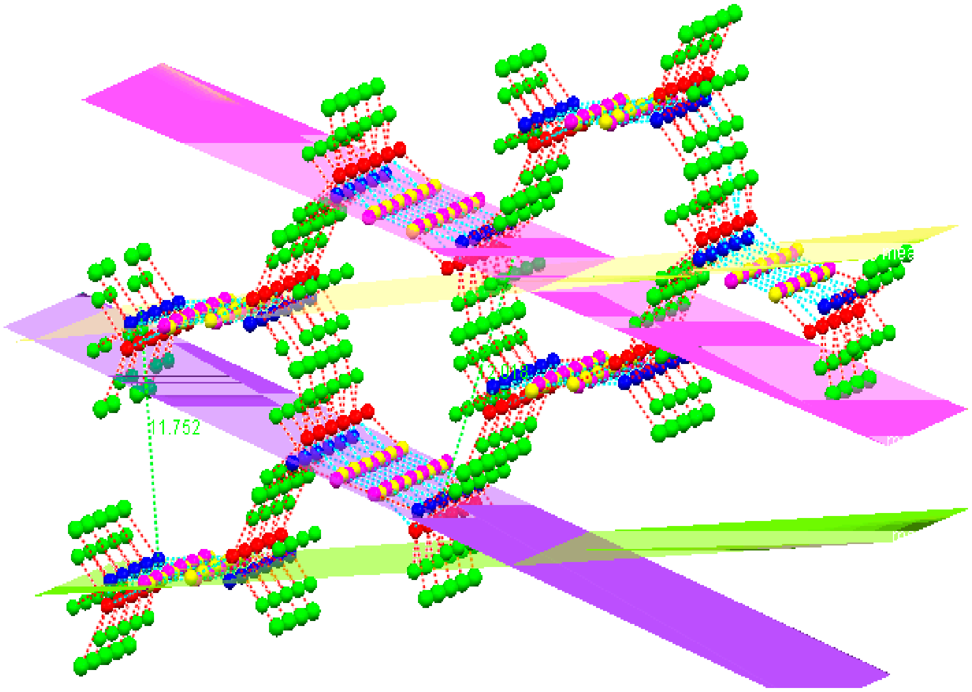

Fig. S6. The distances of the parallel water cluster plane.

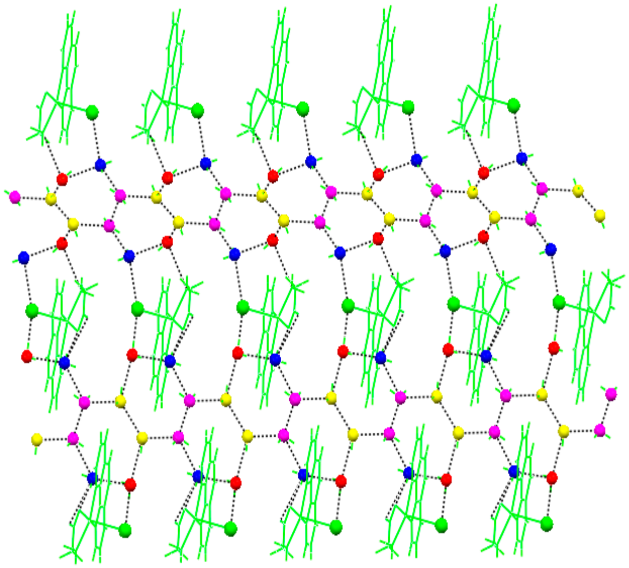

Fig. S7 Depicting hydrogen bond interaction of [CuCl(phen)(gly)] with the water cluster. Water cluster and Cl atom are ball and stick model for clarity. Colours are as follows: blue, O1w; red, O2w; pink, O3w; yellow, O4w; black dashed, hydrogen bond; green and capped stick, [CuCl(phen)(gly)] group and hydrogen atom.

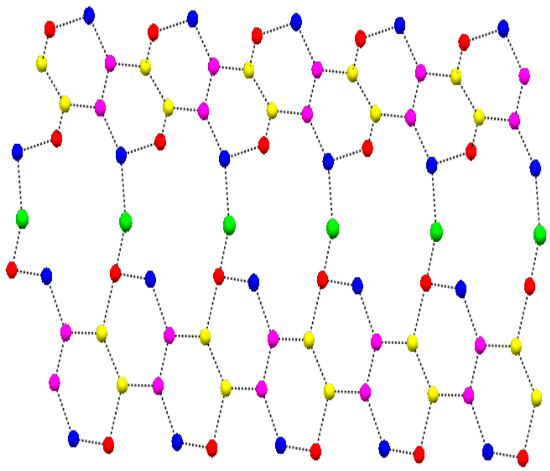

Fig. S8 View of the water–chloride T12(3) aggregate. Colours are as same as **Fig. S7**.

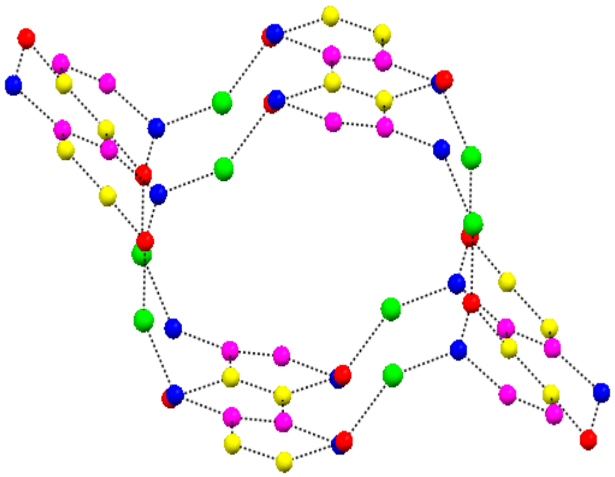

Fig. S9 The formation of water cluster cavity. Cl atom are detained and [Cu(phen)(gly)] are omitted for clarity. Colours are as follows: blue, O1w; red, O2w; pink, O3w; yellow, O4w; Green, Cl atom.

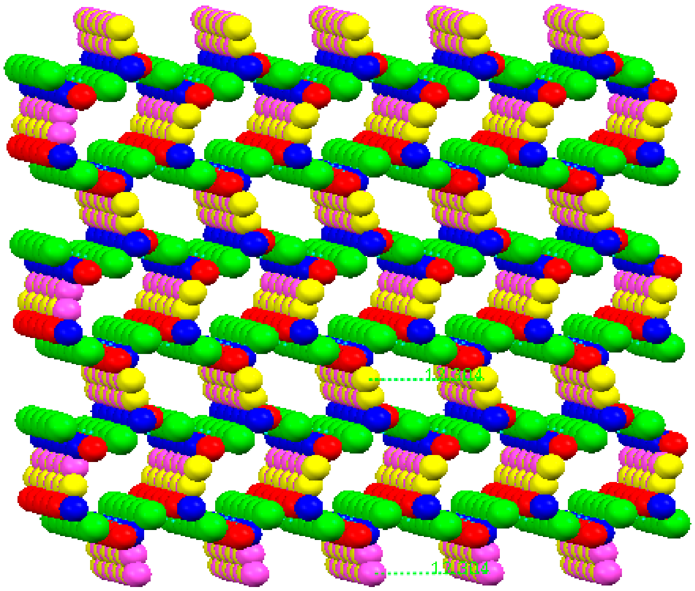

Fig. S10. View of the nanosized water cluster conduit down the *a* axis. Chloride atoms are detained, and [CuCl(phen)(gly)] group are omitted for clarity. Colours are as follows: blue, O1w; red, O2w; pink, O3w; yellow, O4w; green, Cl.

3. Crystallographic data for the compound 1

Table S1. Crystal data and structure refinement

Empirical formula	C14 H20 Cl Cu N3 O6
Formula weight	425.32
Temperature	293(2) K
Wavelength	0.71073 Å
Crystal system, space group	Monoclinic, P21/c
Unit cell dimensions	a = 7.011(2))Å α= 90 deg.<br>b = 12.304(3)Å β= 104.31(3) deg.<br>c = 20.878(5) Å γ= 90 deg.
Volume	1744.9(7)$Å^3$
Z, Calculated density	4, 1.619Mg/m^3
Absorption coefficient	1.441 mm^{-1}
F(000)	876
Crystal size	0.35 x0.28 x 0.20 mm
Theta range for data collection	1.94 to 25.00 deg.
Limiting indices	-8<=h<=8, -14<=k<=12, -24<=l<=17
Reflections collected / unique	6920 / 3066 [R(int) = 0.0296]
Completeness to theta = 25.0	99.7 %
Refinement method	Full-matrix least-squares on F^2
Data / restraints / parameters	3066 / 0 / 226
Goodness-of-fit on F^2	1.356
Final R indices [I>2sigma(I)]	R1 = 0.0717, wR2 = 0.1519
R indices (all data)	R1 = 0.0750, wR2 = 0.1533
Extinction coefficient	0.0053(17)
Largest diff. peak and hole	0.490 and -0.419 e. $Å^{-3}$

4. Table of bond lengths and angles

Table S2. Bond lengths [Å] and angles [deg]

Cu(1)-O(1)	1.950(4)	O(1)-Cu(1)-N(3)	83.80(1)
Cu(1)-N(3)	2.000(5)	O(1)-Cu(1)-N(1)	92.50(1)
Cu(1)-N(1)	2.021(4)	N(3)-Cu(1)-N(1)	164.2(2)
Cu(1)-N(2)	2.028(5)	O(1)-Cu(1)-N(2)	167.4(2)
Cu(1)-Cl(1)	2.577(2)	N(3)-Cu(1)-N(2)	98.60(2)
O(2)-C(13)	1.225(7)	N(1)-Cu(1)-N(2)	82.00(2)
O(1)-C(13)	1.280(7)	O(1)-Cu(1)-Cl(1)	94.80(2)
N(1)-C(1)	1.322(7)	N(3)-Cu(1)-Cl(1)	98.70(2)
N(1)-C(12)	1.353(7)	N(1)-Cu(1)-Cl(1)	97.00(2)
N(2)-C(10)	1.326(7)	N(2)-Cu(1)-Cl(1)	97.00(1)
N(2)-C(11)	1.367(6)	C(13)-O(1)-Cu(1)	115.6(4)
N(3)-C(14)	1.475(7)	C(1)-N(1)-C(12)	119.0(5)
C(1)-C(2)	1.408(8)	C(1)-N(1)-Cu(1)	128.7(4)
C(2)-C(3)	1.358(9)	C(12)-N(1)-Cu(1)	112.2(4)
C(3)-C(4)	1.402(9)	C(10)-N(2)-C(11)	117.7(5)
C(4)-C(12)	1.418(7)	C(10)-N(2)-Cu(1)	130.1(4)
C(4)-C(5)	1.419(9)	C(11)-N(2)-Cu(1)	112.1(4)
C(5)-C(6)	1.356(9)	C(14)-N(3)-Cu(1)	107.9(4)

5. Table of Geometrical Parameters of Hydrogen Bonds for the Water Cluster

Table S3. Geometrical Parameters of Hydrogen Bonds (Å, deg) for the Water Cluster

length		angle	
O1w-O1	2.920(1)	$O2w^{F}$…O1w…$O3w^{C}$	114.39
O1w-O2	3.005(1)	$O2w^{F}$…O1w…O1	84.88
O1w-$Cl(1)^{A}$	3.080(2)	$O2w^{F}$…O1w…O2	109.56
O1w-$O3w^{C}$	2.757(1)	$O2w^{F}$…O1w…$Cl(1)^{A}$	99.34
O2w-$O1w^{B}$	2.743(1)	$O3w^{C}$…O1w…O1	96.28
O2w-$O4w^{C}$	2.754(1)	$O3w^{C}$…O1w…O2	115.53
N3-$O2w^{C}$	3.043	$O3w^{C}$…O1w…$Cl(1)^{A}$	126.05
$O2w^{B}$-Cl(1)	3.223(1)	$O1w^{B}$…O2w…$O4w^{C}$	98.83
O3w-O4w	2.403(1)	$O1w^{B}$…O2w…$N3^{C}$	94.22
O3w-$O3w^{D}$	2.412(1)	$O1w^{B}$…O2w…$Cl(1)^{B}$	103.79
O4w-$O4w^{E}$	2.608(1)	$O4w^{C}$…O2w…$N3^{C}$	91.39
		$O4w^{C}$…O2w…$Cl(1)^{B}$	140.25
		$N3^{C}$…O2w… $Cl(1)^{B}$	110.64
		O4w…O3w…$O3w^{D}$	108.76
		O4w…O3w…$O1w^{G}$	118.00
		$O3w^{D}$…O3w…$O1w^{G}$	132.81
		O3w…O4w…$O4w^{E}$	122.81
		O3w…O4w…$O2w^{G}$	107.92
		$O4w^{E}$…O4w…$O2w^{G}$	127.86
		N3-H3C…$O2w^{G}$	136.87
		O1w-H11w…O1	171.01
		O1w-H11w…O2	118.63
		O2w-H12w…$O1w^{B}$	149.01
		O3w-H13w…$O1w^{G}$	142.37
		O4w-H14w…$O2w^{G}$	156.68
		O1w-H21w…$O3w^{C}$	101.93
		O2w-H22w…$Cl(1)^{B}$	155.70

Symmetry code: A, -x, -1/2+y, 1/2-z; B, 1+x, y, z; C, 1-x, -1/2+y, 1/2-z; D, 1-x, 1-y, -z; E, -x, 1-y, z; F, -1+x, y, z; G, 1-x, 1/2+y, 1/2-z.

6. Table of *π-π* Interactions (Face-to-Face)

Table S4. *π-π* Interactions (Face-to-Face) in complex 1[a]

ring(*i*)→ring(*j*)/C	distance between the (*i,j*) ring centroids(Å)	dihedral angle (*i,j*) (deg)	distance of centroid(*i*) from ring (*j*) (Å)
R1→R4[i]	3.528	0.67	3.354
R2→R3	3.618	2.10	3.379
R2→R4[ii]	3.844	0.92	3.372
R2→R4[i]	3.588	0.92	3.379
R4→R4[ii]	3.528	0.67	3.389
R4→R4[i]	3.865	0.00	3.389

[a] Symmetry code: (i)= -x,1 -y,1-z; (ii)=1-x, 1-y, 1-z.

R(i)/R(j) denotes the *i*th/*j*th rings of phen: R(1)=Cu(1)/N(1)/C(12)/C(11)/N(2); R(2)=N(1)/C(1)/C(2)/C(3)/C(4)/C(12);R(3)=N(2)/C(10)/C(9)/C(8)/C(7)/C(11);R(4)=C(4)/C(5)/C(6)/C(7)/C(11)/C(12).
